# Impact of Surgery and Chemotherapy on Metastatic Extrauterine Leiomyosarcoma

**DOI:** 10.3390/curroncol29040187

**Published:** 2022-03-26

**Authors:** Yoshinori Imura, Satoshi Takenaka, Hidetatsu Outani, Takaaki Nakai, Naohiro Yasuda, Sho Nakai, Toru Wakamatsu, Hironari Tamiya, Seiji Okada

**Affiliations:** 1 Department of Orthopaedic Surgery, Osaka University Graduate School of Medicine, 2-2 Yamadaoka Suita, Osaka 565-0871, Japan; h-otani@ort.med.osaka-u.ac.jp (H.O.); taknakai000m@gmail.com (T.N.); o9o5o5442o4@gmail.com (N.Y.); seokada@ort.med.osaka-u.ac.jp (S.O.); 2 Department of Musculoskeletal Oncology Service, Osaka International Cancer Institute, 3-1-69 Otemae, Osaka 541-8567, Japan; s.takenaka.0816@gmail.com (S.T.); s.nakai.0925@gmail.com (S.N.); evolutionhhh49@yahoo.co.jp (T.W.); tamiyahironari@yahoo.co.jp (H.T.)

**Keywords:** metastatic extrauterine leiomyosarcoma, overall survival, metastasectomy, chemotherapy

## Abstract

Background: Few studies have described the characteristics and prognostic factors of patients with metastatic extrauterine leiomyosarcoma (euLMS). Therefore, we retrospectively investigated the clinicopathological features, clinical outcomes, and prognostic factors of patients with euLMS. Methods: We recruited 61 patients with metastatic euLMS treated from 2006 to 2020 and collected and statistically analyzed information on patient-, tumor-, and treatment-related factors. The median follow-up period was 21.1 months. Results: Sixty-one patients with euLMS and a median age of 59 years were included. Furthermore, their five-year overall survival (OS) rate was 38.3%. Univariate analysis revealed that primary tumor size >10 cm, synchronous metastasis, initial metastatic sites >1, and no metastasectomy with curative intent were significantly associated with poor OS rate. Multivariate analysis identified primary tumor size >10 cm as an independent prognostic factor for poor OS. Among 24 patients who received metastasectomy with curative intent, the interval from the initial diagnosis to development of metastasis ≤6 months was significantly correlated with unfavorable OS. Among 37 patients who did not receive metastasectomy, chemotherapy after metastasis development was significantly related to better OS. Conclusions: Complete metastasectomy should be considered for metastatic euLMS treatment. Moreover, chemotherapy could prolong survival in patients with metastasis who are ineligible for metastasectomy.

## 1. Introduction

Leiomyosarcoma (LMS) represents a heterogeneous subset of soft tissue sarcomas (STSs). Additionally, LMS is a malignant mesenchymal tumor originating from smooth muscle tissues and accounts for 5–10% of all newly diagnosed STSs [1,2,3,4]. LMS is commonly diagnosed in the fifth and sixth decades of life, and it can appear at almost all anatomic sites, such as the uterus, retroperitoneum, extremities, and blood vessels.

Surgical resection is the cornerstone treatment for patients with localized LMS, independent of the origin site. The standard surgical procedure involves a complete excision with wide negative margins, offering the best chance of cure. Performing a complete surgical resection at the initial presentation is the most important prognostic factor for survival. Despite this optimal local treatment, the rate of metastatic relapse is approximately 40% [4,5,6]. Furthermore, metastasis can be present at diagnosis or arise during treatment and follow-up. Prognosis is poor in the metastatic setting, with overall survival (OS) ranging from 10 to more than 30 months [4,5,6,7,8,9,10].

Two primary categories can be distinguished, uterine LMS (uLMS) and extrauterine LMS (euLMS) [11]. uLMS is the most common subtype of uterine sarcoma, likely accounting for the single largest site-specific LMS group [12]. First, gene expression profiling studies suggest a small difference between uLMS and euLMS [13]. Second, several studies suggest that uLMS differs in sensitivity to chemotherapy compared with other STS subtypes [14]. Finally, factors influencing the prognosis for patients with metastatic euLMS are not well described, and limited data regarding responses to systemic therapy are available.

This retrospective study investigates the clinicopathological features, clinical course, treatment outcomes, and prognostic factors in patients with metastatic euLMS treated at our institutions.

## 2. Materials and Methods

We designed a two-institutional retrospective study conducted in Osaka University Hospital and Osaka International Cancer Institute. We collected clinical and pathologic information for patients with metastatic euLMS treated at our institutions between January 2006 and December 2020. Patients’ eligibility criteria included metastatic euLMS diagnosis, pathologically confirmed by a musculoskeletal tumor pathologist at each institution. The Institutional Review Board approved this study.

Sixty-one patients with metastatic euLMS treated at our hospitals were included in this study. The median follow-up period was 21.1 months (range, 1.8–158.8 months). Information on patient-related factors (age and sex), tumor-related factors (site of primary lesions; tumor size, depth, and histological grade; metachronous or synchronous metastasis; duration from the date of initial diagnosis to that of metastasis development; and the number of initial metastatic sites and lesions), treatment-related factors (surgery of the primary tumor and metastatic lesions and chemotherapy and radiotherapy status), local and distant relapse, follow-up period, and oncological outcome at final follow-up were anonymously collected from patients’ medical charts. Synchronous metastasis was defined as that presenting simultaneously as the primary tumor diagnosis. In contrast, metachronous metastasis was defined as that developing after completion of the initial curative treatment. Unfortunately, we could not obtain data on tumor grade in three patients who received their first surgeries at other hospitals.

Objective responses to chemotherapy were determined by Response Evaluation Criteria in Solid Tumors, v.1.1 (RECIST v.1.1). ORR (objective response rate) was defined as the proportion of confirmed complete response (CR) or partial response (PR) to the best response. However, DCR (disease control rate) was defined as the percentage of confirmed CR, PR, or stable disease (SD) to the best response. We calculated the OS rate from the date of metastasis diagnosis to death from any cause or the last follow-up visit. Furthermore, progression-free survival (PFS) was defined as the duration from the date of chemotherapy initiation to that of radiographic progressive disease (PD), discontinuation due to adverse events, death from any cause, or the last follow-up visit. In addition, we calculated the OS rate and PFS using the Kaplan–Meier method and evaluated the impact of prognostic factors using the log-rank test in a univariate analysis. We conducted multivariate analysis using the Cox proportional-hazards model, with variables chosen using a forward conditional stepwise approach. Hazard ratios (HR) were listed with their 95% confidence intervals (CIs). Differences were considered significant when *p*-values were <0.05. EZR software (Saitama Medical Center, Jichi Medical University, Saitama, Japan), a graphical user interface for R (The R Foundation for Statistical Computing, Vienna, Austria), was used for statistical analyses.

## 3. Results

### 3.1. Patient-, Tumor-, and Treatment-Related Characteristics

Patient-, tumor-, and treatment-related characteristics of the 61 cases are presented in Table 1. The 25 male (41%) and 36 female (59%) patients had a median age of 59 years (range, 25–85 years) at metastatic disease diagnosis. Thirty-four patients (55.7%) were ≤60 years, and 27 patients (44.3%) were >60 years.

Sites of primary lesions were the extremities in 22 (36.1%), trunk in 9 (14.8%), the retroperitoneum in 17 (27.9%), and others in 13 (21.3%) patients. The tumor size was ≤10 cm in the greatest dimension in 37 patients (60.7%) and >10 cm in 24 patients (39.3%), with a median size of 8 cm (range, 2.3–24 cm). The tumor depth was categorized as either superficial or deep in the investing fascia. Ten patients (16.4%) had superficial tumors, and fifty-one (83.6%) had deep tumors. Furthermore, we determined the histological grade using the Fédération Nationale des Centers de Lutte Contre le Cancer (FNCLCC) grading system [15]. Twenty-five patients (43.1%) had FNCLCC Grade 2 tumors, and thirty-three (56.9%) had FNCLCC Grade 3 tumors. In addition, 41 patients (67.2%) had metachronous metastases and 20 (32.8%) developed synchronous metastases. For patients with metachronous metastases, the median interval between initial diagnosis and metastatic relapse was 14.7 months (range, 2.1–108.2 months). For example, 48 patients (78.7%) had a single initial metastatic site, and 13 (21.3%) had multiple initial metastatic sites. The most common sites of initial metastases were the lungs (38 patients, 62.3%), followed by the liver (10 patients, 16.4%), muscle (10 patients, 16.4%), lymph nodes (7 patients, 11.5%), and bones (6 patients, 9.8%). Twenty patients (32.8%) had a single metastatic lesion, and 41 patients (67.2%) had multiple metastatic lesions at metastasis diagnosis.

Additionally, fifty-three patients (86.9%) underwent surgery on the primary tumor, and the remaining eight (13.1%) could not undergo surgery of the primary tumor due to metastases at the first visit and inoperative local conditions for surgical treatment. Among 41 patients with metachronous metastases who underwent surgical resection of their primary tumors, ten (24.4%) received neoadjuvant or adjuvant (or both) chemotherapy. In addition, nine patients underwent doxorubicin plus ifosfamide regimens. Thirty patients (49.2%) underwent metastasectomy irrespective of the anatomical site of the metastases. In addition, twenty-four patients (39.3%) underwent complete resection of metastatic lesions, defined as metastasectomy with curative intent. The most common initial metastatic sites where metastasectomy with curative intent was performed were lungs (13 patients), followed by the liver (4 patients), muscle (4 patients), lymph nodes (2 patients), and skin (1 patient).

Chemotherapy and radiotherapy for metastatic lesions were given to 48 (78.7%) and 24 (39.3%) patients, respectively. Various chemotherapy regimens, including doxorubicin, doxorubicin plus ifosfamide, gemcitabine plus docetaxel, pazopanib, trabectedin, and eribulin, were administered. Furthermore, the median number of chemotherapy regimens for patients with metastatic euLMS was 2 (range, 1–5). Of the patients evaluable for response, data for best response, ORR, DCR, and median PFS are described in Table 2.

### 3.2. Survival and Outcomes

At the final follow-up, 12 patients (19.7%) had no evidence of the disease; however, 15 (24.6%) were alive with the disease, and 34 (55.7%) died of the disease. The five-year OS rate of all patients with metastatic euLMS was 38.3%, with a median OS period of 30.7 months (range, 1.8–158.8 months). Furthermore, among 53 patients who underwent surgery of the primary tumor, local recurrence developed in 11 (20.8%). Finally, surgical removal of the local recurrent tumor was performed in five patients.

### 3.3. Prognostic Factor Analyses

**Figure 1:**
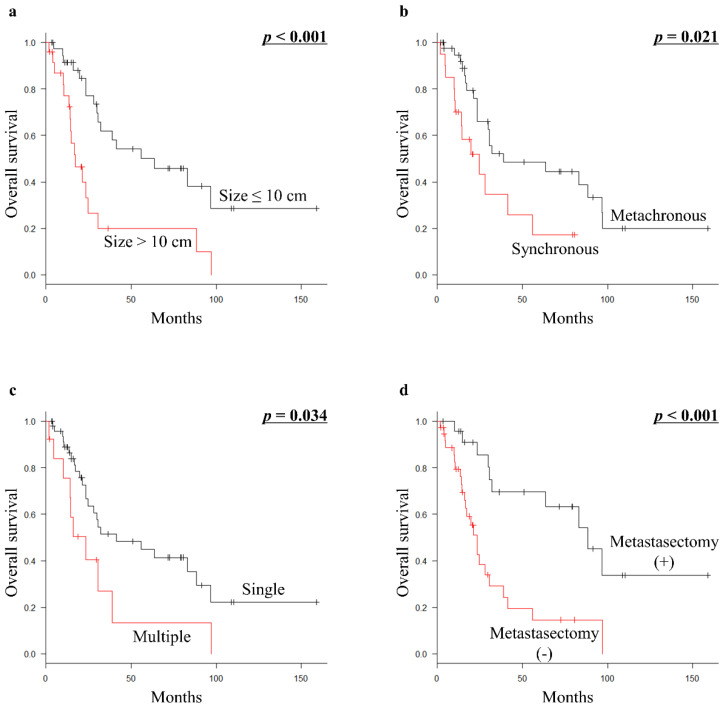
Kaplan–Meier survival curves in all 61 patients with metastatic euLMS: (**a**) OS according to tumor size (≤10 cm vs. >10 cm). (**b**) OS according to presenting status (metachronous vs. synchronous). (**c**) OS according to the number of initial metastatic sites (single vs. multiple). (**d**) OS according to metastasectomy with curative intent (presence vs. absence of metastasectomy with curative intent). euLMS, extrauterine leiomyosarcoma; OS, overall survival.

For all 61 patients with metastatic euLMS, primary tumor size (*p* < 0.001), presenting status at initial diagnosis (*p* = 0.021), number of initial metastatic sites (*p* = 0.034), and metastasectomy with curative intent (*p* < 0.001) were significant prognostic factors for OS in univariate analyses (Table 1; Figure 1a–d). However, multivariate analysis revealed that primary tumor size >10 cm (HR 2.48; 95% CI 1.137–5.411; *p* = 0.023) was a significant prognostic factor for unfavorable OS in all patients (Table 3).

**Figure 2:**
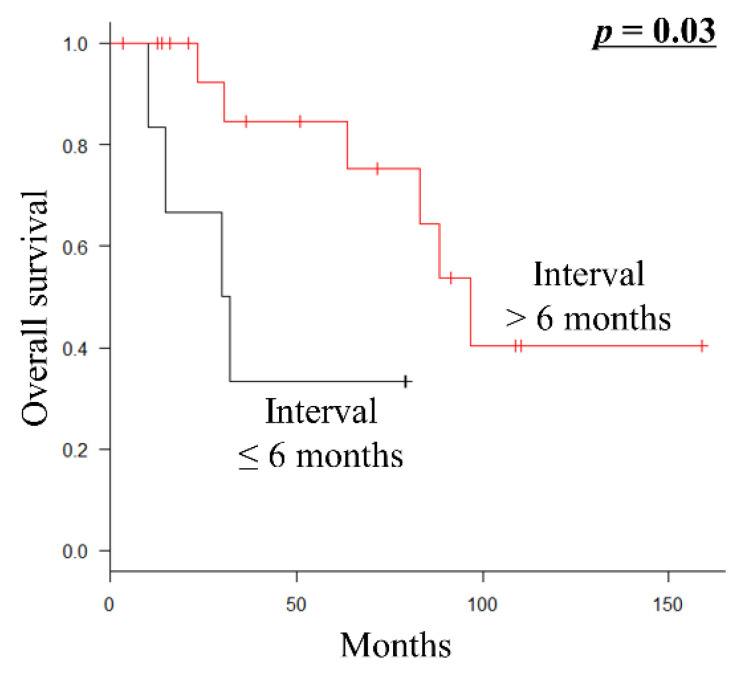
Kaplan–Meier survival curve of OS according to the interval from the initial diagnosis to metastasis (≤6 months vs. >6 months) in 24 patients with metastatic euLMS who underwent metastasectomy with curative intent. OS, overall survival; euLMS, extrauterine leiomyosarcoma.

Of the 24 patients who underwent metastasectomy with curative intent, 23 (95.8%) presented with metachronous metastatic disease. Furthermore, all patients undergoing metastasectomy with curative intent had undergone surgical resection of the primary tumor before metastasectomy. Therefore, they had one or two metastatic lesions at a single initial metastatic site. Fourteen patients (58.3%) received systemic chemotherapy before and/or after metastasectomy. By univariate analysis, the interval from the initial diagnosis to metastasis development ≤6 months (*p* = 0.03) was a significantly poor prognostic factor for OS (Table 4; Figure 2).

**Figure 3:**
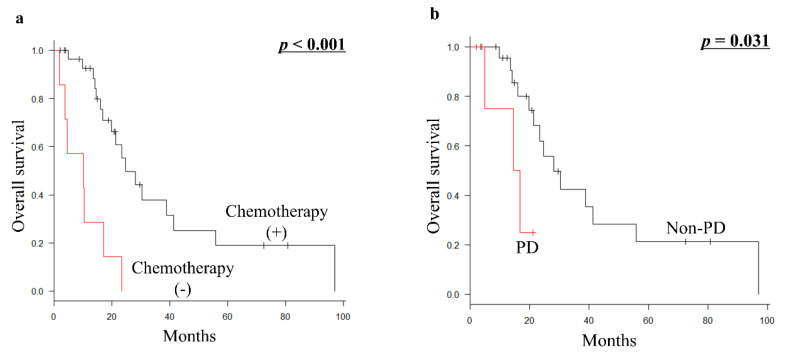
(**a**) Kaplan–Meier survival curve of OS according to palliative chemotherapy (presence vs. absence of chemotherapy) in 37 patients with metastatic euLMS who were ineligible for metastasectomy with curative intent. (**b**) Kaplan–Meier survival curve of OS according to the response of first-line chemotherapy from developing metastasis (non-PD vs. PD) in 30 patients with metastatic euLMS who were ineligible for metastasectomy with curative intent and received palliative chemotherapy. OS, overall survival; euLMS, extrauterine leiomyosarcoma; PD, progressive disease.

Palliative chemotherapy (*p* < 0.001) was significantly associated with a better prognosis for OS in 37 patients who were ineligible for metastasectomy with curative intent (Table 5; Figure 3a). Furthermore, among 30 patients who received palliative chemotherapy, the most common first-line chemotherapy regimens were doxorubicin plus ifosfamide (10 patients), followed by gemcitabine plus docetaxel (8 patients), doxorubicin (7 patients), pazopanib (2 patients), eribulin (2 patients), and trabectedin (1 patient). In addition, the prognosis of 24 patients who had non-PD (PR or SD) during first-line palliative chemotherapy (*p* = 0.031) was significantly better than that of 6 patients who had PD (Figure 3b).

The median OS periods for patients with metastatic euLMS diagnosed from 2006 to 2013 and those diagnosed from 2014 to 2020 were 36.8 and 29.9 months, respectively. There was no significant difference between the two groups (*p* = 0.742).

## 4. Discussion

A single-center, retrospective review of 353 patients with primary euLMS identified size and grade as distinct factors influencing disease-specific survival [16]. However, there are relatively little data published regarding factors influencing survival in patients with metastatic euLMS. In our study, the median OS from the diagnosis of metastatic disease was 30.7 months, which is higher than the data reported by others [4,5,6,7,8,9,10]. Furthermore, univariate analyses of our cohort showed that primary tumor size, presenting status at initial diagnosis, number of initial metastatic sites, and metastasectomy with curative intent were associated with significant differences in OS. We identified primary tumor size >10 cm as an independent risk factor for decreased OS in the metastatic euLMS population. The histological tumor grade was not significantly associated with survival in univariate analysis, suggesting that tumor grade did not affect OS after metastasis development.

Surgical resection of primary tumors is considered the primary local treatment for patients with LMS and localized disease, prolonging their survival [17]. However, treating metastatic LMS remains a challenge, as a curative treatment for metastatic disease is rare. The appropriate treatment for patients with metastatic euLMS remains unknown. In our cohort, surgical resection of primary tumors was performed in 86.9% of all patients with metastatic euLMS but did not significantly prolong their survival. Patients with metastatic LMS of any site should be evaluated to determine whether the resection of metastases may be appropriate. Additionally, favorable five-year survival rates of 38%–52% following pulmonary metastasectomy for LMS have been reported [18,19,20]. In this study, the five-year OS rate of patients with metastatic euLMS who received metastasectomy with curative intent was 69.5%. They had received surgical resection of primary tumors before metastasectomy, and most of them had metachronous metastatic disease. These results suggest that primary and metastatic lesions should be actively treated to obtain maximum survival time.

Additionally, resection should be considered for patients with a relatively long disease-free interval and an isolated disease site amenable to complete resection, with an acceptably low risk of morbidity. However, in our study, all patients undergoing metastasectomy with curative intent had one or two metastatic lesions at a single initial metastatic site. The extended disease-free interval following resection of a primary tumor to the occurrence of pulmonary metastases has also been demonstrated by several authors to be a positive predictor of survival. The best results are seen with a disease-free interval of 12 months or longer [21]. In this study, the interval from the initial diagnosis to development of metastasis ≤6 months but not 12 months was significantly associated with unfavorable OS among patients who received metastasectomy with curative intent. However, this finding could be the result of the patient selection process. Therefore, establishing the benefit of metastasectomy would require further randomized clinical trials.

Different STS subtypes have recognized variable patterns of chemosensitivity, and LMS shows moderate sensitivity to chemotherapy. Doxorubicin-based chemotherapy is commonly used to treat patients with metastatic STS, alone or with ifosfamide [22]. Combining gemcitabine plus docetaxel, unlike other STSs, seems to be effective in LMS as first- and second-line treatments in patients who have previously received doxorubicin-based therapy. Furthermore, in the phase III multicenter trial, gemcitabine and docetaxel vs. doxorubicin as a first-line treatment in previously untreated advanced unresectable or metastatic soft tissue sarcoma (GeDDiS) did not observe differences in response rate and PFS from first-line gemcitabine plus docetaxel treatment compared with single-agent doxorubicin, with both regimens demonstrating activity in LMS [23]. Therefore, regimens to consider for first-line therapy include doxorubicin-based therapies and gemcitabine plus docetaxel. In this study, the main reasons for avoiding doxorubicin-based regimens were advanced age and cardiac dysfunction.

Several regimens have shown activity in LMS as second-line treatment or later. Since 2012, three anticancer drugs, pazopanib, trabectedin, and eribulin, have been approved in Japan for the second-line or later treatment of patients with advanced STS of any histologic subtype, including LMS. In the randomized phase III study (the PALETTE trial) in 372 patients with advanced non-adipocytic STS, pazopanib improved PFS compared with placebo in STS. However, there was no difference in OS, and ORR was observed in only 4% of patients [24]. Subgroup analysis by histologic subtype and predictive analysis for histology subtype using Cox models showed pazopanib to be effective for LMS in terms of PFS [24,25]. The phase III trial of trabectedin demonstrated trabectedin superiority over dacarbazine in PFS, but not in ORR or OS [26]. Several studies, including data from 431 patients with LMS of any origin treated in a trabectedin-expanded access program, demonstrated an ORR of 7.5% in patients with LMS compared with 5.9% among patients with all-type STS [27]. In the phase III trial of eribulin for L-sarcomas, eribulin demonstrated superior OS benefit than dacarbazine, but this was not the case for PFS or response rate [28]. Subgroup analysis by histologic subtype of the data for OS showed the effect of eribulin on LMS to be similar to that of dacarbazine [29].

In our cohort, palliative chemotherapy for patients with metastatic euLMS ineligible for metastasectomy proved to be of significant prognostic value for better OS. This suggests that palliative chemotherapy may help prolong the survival of these patients. Patients with PR or SD to first-line chemotherapy had significantly better OS than patients whose tumors showed PD regardless of the type of chemotherapy used. It is hoped that biomarkers of response that will help to optimize treatment choices for patients with LMS may be identified.

We believe that our study shows the efficacy of surgery and chemotherapy in patients with metastatic euLMS. There are several limitations to this study. First, the results of this study must be interpreted with caution due to its retrospective design and limited sample size. Second, we could not obtain precise histological grading information in three patients who received surgical resection of the primary tumor in other hospitals. Third, there was possible selection bias concerning receipt of surgery and chemotherapy because frail patients with limited life expectancy are often not offered aggressive multimodality treatment. Fourth, each physician decided the choice of drugs used. During the study period, we could use pazopanib, trabectedin, and eribulin since 2012, 2015, and 2016, respectively. These limitations should be considered when evaluating the results of this study. Therefore, further investigations, including a prospective randomized study, are needed.

## 5. Conclusions

The five-year OS rate of patients with metastatic euLMS was 38.3%. Large primary tumor size was significantly associated with poor OS in multivariate analysis. Therefore, complete metastasectomy should be performed for patients with metastatic euLMS whose primary tumors were resected. Moreover, palliative chemotherapy could prolong survival in patients who are ineligible for metastasectomy. A multidisciplinary approach for metastatic euLMS is necessary, and, thus, understanding how to select the best therapies that may benefit patients with advanced euLMS is important.

## Figures and Tables

**Table 1:** Patient-, tumor-, and treatment-related characteristics and univariate analysis of prognostic factors for OS in 61 patients with metastatic euLMS. OS, overall survival; euLMS, extrauterine leiomyosarcoma; N.A., not available.

Factors		N (%)	Median OS (Months)	*p* Value
Age	≤60	34 (55.7)	38.8	0.175
	>60	27 (44.3)	23.3	
Sex	Male	25 (41)	23.3	0.112
	Female	36 (59)	55.9	
Primary site	Extremity	22 (36.1)	29.9	0.502
	Trunk	9 (14.8)	83	
	Retroperitoneum	17 (27.9)	30.4	
	Others	13 (21.3)	28.2	
Size	≤10 cm	37 (60.7)	55.9	<0.001
	>10 cm	24 (39.3)	17.1	
Depth	Superficial	10 (16.4)	N.A.	0.093
	Deep	51 (83.6)	29.9	
Grade	2	25 (43.1)	41.3	0.244
	3	33 (56.9)	23.3	
	N.A.	3	-	-
Presenting status	Metachronous	41 (67.2)	38.8	0.021
	Synchronous	20 (32.8)	24.7	
Number of initial metastatic sites	1	48 (78.7)	41.3	0.034
	>1	13 (21.3)	23.4	
Resection of primary tumor	Yes	53 (86.9)	32.2	0.071
	No	8 (13.1)	24.7	
Metastasectomy with curative intent	Yes	24 (39.3)	88.3	<0.001
	No	37 (60.7)	23.3	
Chemotherapy	Yes	48 (78.7)	32.2	0.917
	No	13 (21.3)	23.3	
Radiotherapy	Yes	24 (39.3)	30.7	0.91
	No	37 (60.7)	29.9	

**Table 2:** Best response, ORR, DCR, and PFS of chemotherapy regimens. CR, complete response; PR, partial response; SD, stable disease; PD, progressive disease; ORR, objective response rate; DCR, disease control rate; PFS, progression-free survival; DXR, doxorubicin; IFM, ifosfamide; GEM, gemcitabine; DOC, docetaxel; PAZ, pazopanib; TRB, trabectedin; ERB, eribulin.

Regimen	N	Best Response				ORR (%)	DCR (%)	Median PFS (Months)
		CR	PR	SD	PD			
DXR	9	0	1	6	2	11.1	77.7	4.9
DXR + IFM	18	1	4	8	5	27.8	72.2	6.1
GEM + DOC	22	0	7	8	7	31.8	68.2	4.5
PAZ	24	0	1	14	9	4.2	62.5	3.5
TRB	9	0	0	4	5	0	44.4	2.1
ERB	14	0	1	8	5	7.1	64.3	3.5

**Table 3:** Multivariate analysis of prognostic factors for OS in 61 patients with metastatic euLMS. OS, overall survival; euLMS, extrauterine leiomyosarcoma; HR, hazard ratio; CI, confidence interval.

Factors	Multivariate Analysis		
	HR	95% CI	*p* Value
Size >10 cm	2.48	1.137–5.411	0.023
	1		
Synchronous metastasis	1.756	0.701–4.4	0.23
	1		
Initial metastatic sites > 1	1.039	0.385–2.803	0.94
	1		
No metastasectomy	2.236	0.773–6.471	0.138
	1		

**Table 4:** Patient-, tumor-, and treatment-related characteristics and univariate analysis of prognostic factors for OS in 24 patients with metastatic euLMS who underwent metastasectomy with curative intent. OS, overall survival; euLMS, extrauterine leiomyosarcoma; N.A., not available.

Factors		N (%)	Median OS (Months)	*p* Value
Age	≤60	13 (54.2)	96.8	0.208
	>60	11 (45.8)	88.3	
Sex	Male	9 (37.5)	32.2	0.211
	Female	15 (62.5)	96.8	
Primary site	Extremity	10 (41.7)	32.2	0.513
	Trunk	6 (25)	96.8	
	Retroperitoneum	6 (25)	76.05	
	Others	2 (8.3)	30.7	
Size	≤10 cm	19 (79.2)	96.8	0.101
	>10 cm	5 (20.8)	88.3	
Depth	Superficial	7 (29.2)	N.A.	0.289
	Deep	17 (70.8)	83	
Grade	2	10 (45.5)	88.3	0.289
	3	12 (54.5)	83	
	N.A.	2	-	-
Interval from initial diagnosis to metastasis	≤6 months	6 (25)	31.1	0.03
	>6 months	18 (75)	96.8	
Number of initial metastatic lesions	1	18 (75)	88.3	0.888
	2	6 (25)	N.A.	
Chemotherapy prior to or after metastasectomy	Yes	14 (58.3)	83	0.351
	No	10 (41.7)	88.3	
Radiotherapy	Yes	10 (41.7)	83	0.387
	No	14 (58.3)	N.A.	

**Table 5:** Patient-, tumor-, and treatment-related characteristics and univariate analysis of prognostic factors for OS in 37 patients with metastatic euLMS who were ineligible for metastasectomy with curative intent. OS, overall survival; euLMS, extrauterine leiomyosarcoma; N.A., not available.

Factors		N (%)	Median OS (Months)	*p* Value
Age	≤60	21 (56.8)	24.7	0.152
	>60	16 (43.2)	17.1	
Sex	Male	16 (43.2)	19.8	0.205
	Female	21 (56.8)	30.4	
Primary site	Extremity	12 (32.4)	19.8	0.202
	Trunk	3 (8.1)	16.1	
	Retroperitoneum	11 (29.7)	24.7	
	Others	11 (29.7)	28.2	
Size	≤10 cm	18 (48.6)	28.2	0.087
	>10 cm	19 (51.4)	17.1	
Depth	Superficial	3 (8.1)	16.1	0.696
	Deep	34 (91.9)	23.3	
Grade	2	15 (41.7)	24.7	0.349
	3	21 (58.3)	16.1	
	N.A.	1	-	-
Presenting status	Metachronous	18 (48.6)	23.3	0.477
	Synchronous	19 (51.4)	19.8	
Number of initial metastatic sites	1	24 (64.9)	23.3	0.702
	>1	13 (35.1)	23.4	
Resection of primary tumor	Yes	29 (78.4)	21.3	0.704
	No	8 (21.6)	24.7	
Chemotherapy	Yes	30 (81.1)	24.7	<0.001
	No	7 (18.9)	10.2	
Radiotherapy	Yes	14 (37.8)	24.7	0.989
	No	23 (62.2)	21.3	

## Data Availability

The datasets used in this study are available from the corresponding author on reasonable request.
